# Prospective Asian plants with corroborated antiviral potentials: Position standing in recent years

**DOI:** 10.1186/s43088-022-00218-y

**Published:** 2022-04-05

**Authors:** Sania Ashrafi, Mamunur Rahman, Pollob Ahmed, Safaet Alam, Md. Abid Hossain

**Affiliations:** 1 Department of Pharmacy, BRAC University, Mohakhali, Dhaka, 1212 Bangladesh; 2 Department of Pharmacy, East West University, Aftabnagar, Dhaka, 1212 Bangladesh; 3 Department of Pharmacy, Faculty of Allied Health Sciences, Daffodil International University, Daffodil Smart City, Ashulia, Dhaka, Bangladesh; 4 Department of Pharmacy, State University of Bangladesh, 77 Satmasjid road, Dhanmondi, Dhaka, 1205 Bangladesh

**Keywords:** Antiviral, Asian plants, Complementary medicine, Phytochemicals, Active chemical constituents, HSV, HIV

## Abstract

Viral diseases are extremely widespread infections caused by viruses. Amongst numerous other illnesses, viral infections have challenged human existence severely. Over the history of mankind, new viruses have emerged and presented us with new tests. The range of viral infections varies from familiar infectious diseases such as the common cold, flu, and warts to severe ailments such as AIDS, Ebola, and COVID-19. The world has been racing to find an effective cure for the newly evolving viruses. Toxic effects, non-selectivity, drug resistance, and high price are the most common complications of conventional treatment procedures. Nature is a marvelous source of phytoconstituents with incredible varieties of biological activities. By tradition, medicinal plants have been utilized for the treatment of countless infectious diseases worldwide, some of which contain a broad spectrum of activities. Modern drug discovery and development techniques offer highly efficient separation techniques, inauguration of vector-based schemes where the original infectious virus is cloned to the non-infectious one for antiviral screening targets. The objective of the review was to gather available data on 20 both cultivated and native plants of Asia giving antiviral activities and provide comprehensive information on the phytochemical analysis of the plants and potential antiviral compounds isolated from these plants.

## Background

Herbal medicine, also known as herbalism, is about pharmacognosy and using medicinal plants as a foundation of natural therapy [1]. The most ancient evidence of use of the medicinal plant is dated back to 5000 years ago in Nagpur that consisted of twelve recipes including ingredients as an alkaloid [2]. Nowadays, herbal products are readily available in the market. It has been estimated that around 80% of the Asian and African populations are dependent on herbal medicine in their primary approach to treatment [3]. Although use of herbal treatment may exert adverse effects sometimes, its appeal is increasing day by day [4]. Recently, natural remedies are getting more accepted because randomized clinical trials are conducted on different herbal products and research articles are being published [5]. Most herbal drugs are used to treat mild to moderate diseases, and people are likely to use herbal medicine before starting the conventional therapy while being used mostly in chronic conditions [6–8]. Emergence of viral diseases may significantly affect the morbidity, mortality, and economy of the human population. Alternative treatment approaches are available against viruses [9]. Toxicity and drug resistance are the two most important factors that limit the usage of modern antiviral drugs [10]. Information on the adverse effect of herbal medicine is limited, but generally using natural remedies to treat a disease in a known situation is considered as safe and effective [11]. Due to the assumption of fewer side effects caused by herbal medicine, it is safe to use by comparison with the conventional medicine of synthetic origin [12]. Family tradition, past overall good experiences and not being satisfied with conventional treatment options propelled people to choose herbal medicine as a treatment option [4]. Herbal drugs can be made from whole plants, parts of plants, algae, fungi, extracts, essential oils, fatty oils, juices, and processed exudates of herbal materials [13]. Available dosage forms include herbal soaps, herbal tablets, herbal capsules, herbal creams, decoctions, herbal teas, tinctures, glycerites, oxymels, and ointments [14]. Herbal drugs act against viruses in mechanistic ways as inhibition of gene replication, protein function or virus cell fusion, etc. [14]. In this review, we have discussed the phytochemical constituents of twenty medicinal plants available in Asia focusing on their biological action against viruses. This review article will support the researchers in the future to lead further research regarding medicinal plants focusing on antiviral properties to corroborate the role of herbal sources as a treatment appliance.

## Methodology

In this review article, 20 plants, which have certain antiviral properties along with other pharmacological properties, of Asian native were studied, through different reliable databases. Databases with bibliography such as PubMed, Google Scholar, Science Direct, Springer link, MEDLINE, and Scopus were investigated strenuously, and information like general description of plants, phytochemical analysis, and antiviral activities was assembled. SciFinder databases and PubChem were used to authenticate the vital structures of the selected plant constituents. In this review, ChemDraw (version-20) software was used to draw the chemical structure of the major constituents. Mendeley desktop version 1.19.8 was used to cite and reference the information sources.

## Phytochemistry and antiviral properties of the plants

### *Acacia nilotica* (L.) Delile (Fabaceae)

(Synonym: ***Vachellia nilotica***)

*Acacia nilotica* is a medicinal plant which is used for the treatment of various diseases and is widely distributed throughout the tropical and sub-tropical regions. Different parts of the plant like roots, leaves, bark, gum, flowers, and pods are used for treatment of diseases in different countries [15].

#### Phytochemistry

The plant *A. nilotica* contains gallic acids, catechin, analogs of methyl gallate, quercetin, tannic acid, and various other flavonoid and phenolic acids. It also contains a significant number of polyphenols, proteins, alkaloids, saponins, terpenoids, and polypeptides (Raheel et al. 2013). The qualitative phytochemical studies of different part of plant extract showed that, the bark contains terpenoids, alkaloids, tannins, sterols, saponins, and glycosides; leaves contain tannins, sterols, alkaloids, cardiac glycosides, saponins, and flavonoids; roots contain saponins, terpenes, flavonoids, sterols, phenols, tannins, alkaloids, and anthraquinones; pods contain alkaloids, tannins, carbohydrate, flavonoids, saponins, and sterol; flowers showed occurrence of phenolic compound [15].

#### Antiviral activity

Methanolic extract of the plant is active against two animal viruses: Newcastle Disease and Fowl Pox Viruses [16]. The extract of the leaves of the plant showed in vitro antiviral activity against the Turnip Mosaic Virus [15, 17]. The plant possesses anti-viral potential against Peste des Petits Ruminant’s Virus (PPRV). Significant in vitro inhibition of Hepatitis C virus by *A. nilotica* extract was also observed, and the anti-HIV property might be due to the inhibition of reverse transcriptase enzyme [16].

### *Achyranthes aspera* L. (Amaranthaceae)

*Achyranthes aspera* is called Latjira in Hindi. It is an erect, stiff medicinal plant. The plant is available as weed in whole India, Asia, and many parts of the world such as Central America, Mexico, and Africa [18].

#### Phytochemistry

Phytochemicals include alkaloids, saponins, flavonoids, terpenes, quinones, lignans, polysaccharides, tannins, steroidal glycoside, proanthocyanidin, thiosulfinates, and proteins. Oleanolic acid **(1)** has also been isolated from the plant [19].

#### Antiviral activity

The plant showed strong anti-Herpes viral activities [18, 20]. Another study reveals that the anti-HSV activity of *A. aspera* is attributed to oleanolic acid **(1)** found in the plant [19]. Oleanolic acid can stop HSV at its early replication stage. Therefore, OA **(1)** is regarded as a possible HSV infection candidate OA **(1)** with the action of the anti-Herpes virus estimating increased attention and usage for future research [21].

### *Acyranthes bidentata* Blume (Amaranthaceae)

*Achyranthes bidentata* is a perennial herbaceous plant that is widely distributed and grown in the tropical areas of Asia and Africa. The plant is grown abundantly particularly in China, Vietnam, and Korea [22].

#### Phytochemistry

The plant comprises several chemically active components including triterpenes, saponin, steroidal ketone, polysaccharides, and polypeptides. Furthermore, it includes alkaloids like morphine, strychnine, quinine, flavonoids, iridoids, organic acids, volatile oils [23]. Also, a compound named *Acyranthes bidentata* polysaccharide was isolated from the plant’s roots which has several activities [22].

#### Antiviral activity

*Acyranthes bidentata* polysaccharide when sulfated can show activity against Porcine reproductive and RSV [22].

### *Acorus calamus* L. (Acoraceae)

*Acorus calamus* is a popular traditional Chinese medicinal plant, and its root is historically used to treat neurodegenerative diseases, and for cholera treatment. It also possesses antimicrobial properties [24]. *A. calamus* is a native of central Asia, and eastern Europe is indigenous to the marshes of the mountains of India [25].

#### Phytochemistry

Root of *Acorus calamus* contains Tatanan A **(2)** [25]. At least one hundred eighty-five compounds in the oil of the triploid European *A. calamus* var. calamus, and ninety-three compounds in the oil of the tetraploid Indian *A. calamus* var. angustatus with f-asarone as the major constituent are reported. β-Asarone, methyleugenol, geranylacetate, cis-methylisoeugenol, shyobunone, epishyobunone, β-farnesene, and isoshyobunone, calamenene, asaronaldehyde, acorenone, calamenone, α- and γ-asarone, n-heptanic acid, numerous sesquiterpenes, calamendiol, tannins, starches, soft gums, mucin, resins, 2-allyl-5-ethoxy-4-methoxyphenol, 4-terpineol, epieudesmin, lysidine, borneol, furylethyl ketone, nonanoic acid, spathulenol, 2,2,5,5- tetramethyl-3-hexanol, bornyl acetate, retusin, (9E,12E,15E)-9,12,15-octadecatrien-1-ol, butyl butanoate, galgravin, geranyl acetate, acetic acid, camphor, isoelemicin, sakuranin, ursolic acid, dehydroabietic acid, isoeugenol methylether, acetophenone, apigenin 4’,7-dimethyl ether, linalool, elemicin, dehydrodiisoeugenol, linolenic acid are some of the compounds found in the plant [25].

#### Antiviral activity

*Acorus calamus* shows antiviral effect without any significant cytotoxicity on Dengue Virus [26]. Alcohol extract of the rhizome showed potent antiviral activity against HSV-1 and HSV-2 below cytotoxic concentration [25]. Tatanan A **(2)**, a compound isolated from the plant had a novel antiviral activity against Dengue Virus DENV_2_, and it inhibited the post-translation or early RNA synthesis steps. So, it could be used as an anti- Dengue Virus (DENV) lead compound as well [27]. It also showed activity against HIV-1 reverse transcriptase enzyme [28].

### *Aleurites moluccanus* (L.) Willd. (Euphorbiaceae)

This plant, a flowering tree with around 98 ft high, is commonly known as Kukui, Candlenut tree, or Indian walnut. Kukui nut oil can be extracted from the part of the seed of this plant [29]. It is indigenous to Polynesia, Malaysia, and South Sea Island also occurs in Brazil [30].

#### Phytochemistry

Phytochemical evaluation of *Aleurites moluccana* exerted several types of bioactive secondary metabolites including steroids, triterpenes, coumarins, and flavonoid glycosides such as moluccanin, moretenone, acetil aleuritic acid, moretenol, α-amyrin, β-amyrin, stigmasterol, β-sitosterol-3-β-D-glucopyranoside **(3),** swertisin, and campesterol [31]. Swertisin and 2′′-O-rhamnosylswertisin were also reported from the leaves of *A. moluccana.* Another study also reported the isolation of α -amyrin, β -amyrin, stigmasterol, β -sitosterol, n-hentriacontane, and campesterol from the ethyl acetate and butanol fraction of *A. moluccana* [32]. A phorbol diester, 13-O-myristyl-20-O-acetyl12-deoxyphorbol, hentriacontane (hydrocarbon), 6,7-dimethoxycoumarin, 5,6,7-trimethoxycoumarin and β-sitostenone (phytosterol), 2″-Orhamnosylswertisin have also been isolated from the plant [33].

#### Antiviral activity

*Aleurites moluccana* is used to treat Herpes viral infections as conventional Hawaiian medicine [34]. Dichloromethane fraction of *A. moluccana* leaves and barks also showed prominent antiviral activity which validated the traditional use of this plant against viral infections [35]. Hydroalcoholic extract of the plant has shown significant antiviral activity against r Newcastle Disease Virus (NDV) and Avian Influenza Virus (AIV) H_5_N_1_in a study [36].

*A. moluccana* has also demonstrated significant anti-HIV action. The phytosterol, β-sitosterol-3-β-D-glucopyranoside **(3)**, was found as the first chemically pure active component isolated from the methanol extract to exert anti- HIV action [37].

### *Barleria cristata* L. (Acanthaceae)

*Barleria cristata* is commonly known as Philippine violet. It is a commonly cultivated plant used for ornamental purposes, and it was recently established in Southeast Asia, South China, subtropical and tropical regions of India. It is also used in different ethnomedical systems for the treatment of a wide range of diseases [24].

#### Phytochemistry

Iridoid glycosides such as 6-O-trans-p-coumaroyl-8-O-acetylshanzhiside methyl ester and (its cis isomer as well) were discovered from the plant [38]. Ethanolic extract contains phenylethanoid glycosides which includes β-[(30,40-dihydroxyphenyl)-ethyl]-(4″-O-caffeoyl)- β-D-glucoside (desrhamnosylacteoside), β-[(30,40-dihydroxyphenyl)-ethyl]-(3″,6″-O-L-dirhamnosyl)-(4″-O-caffeoyl)-β-D-glucoside and β-[(30,40dihydroxyphenyl)-ethyl]- (3″-O-L-rhamnosyl)-(4″-O-caffeoyl)-β-D-glucoside (acteoside) (poliumoside) phenylethanoid glycosides [24].

#### Antiviral activity

The iridoid glycosides mentioned above showed potent antiviral activity against RSV attacking infants [38]. Phenylethanoid glycosides present in the plant play an important role in several pharmacological activities including antiviral activity [24].

### *Betula utilis* D.Don (Betulaceae)

Being commonly known as ‘Bhojpatra’ in India, the plant is a perennial, medium-sized tree that expands up to 20 m in height [39] and is well distributed from inner Mongolia north of China to Yunnan province in the south and over the Himalayan region of India, Afghanistan, Bhutan, and Nepal [40].

#### Phytochemistry

Sitosterol, betulinic acid **(4)**, betulin **(5)**, 3-acetyloleanolic acid, oleanolic acid, lupeol, methyl betulonate, lupenone, methyl betulate, and a new triterpenoid karachic acid have been isolated from the bark of the plant. Leucocyanidin and polymeric leucoanthocyanidins are also found in this plant [41]. In another experiment, six triterpenes namely betulinic acid, betulin, ursolic acid, lupeol, oleanolic acid and ß-amyrin have been isolated from the ethyl acetate extract of the plant [42]. The essential oil of *B.utilis* combines seleneol, linalool, champacol, sesquiphellandrene, geranic acid, 1,8-cineole. Fatty acid portion is composed of myristic, linoleic, palmitic and oleic acid [39].

#### Antiviral activity

Betulinic acid **(4)** isolated from the plant has been reported to act against HIV by inhibiting its replication [39, 43]. Derivatives of betulinic acid showed antiviral activity by inhibition of HIV entry [44] and HIV protease [45]. In another study, betulin **(5)** showed antiviral activity against HSV-1 and HSV-2 viruses [46].

### *Bidens pilosa* L. (Asteraceae)

*B. pilosa* is an erect, perennial herb, grows up to 1.5 m, widely distributed across tropical and subtropical region countries. It is commonly known as *xian feng cao* (all bountiful grass) in Chinese tradition. Traditionally, leaves and whole plant parts were used to treat influenza in China, Middle America, and Uganda [47].

#### Phytochemistry

The plant is a reservoir of countless important secondary metabolites like aliphatic natural compounds, saturated unbranched alcohols, saturated unbranched carboxylic acids, unbranched aliphatic carboxylic acid esters, acetylenic hydrocarbons, simple aromatic hydrocarbons, phenylpropanoids, porphyrins, carbohydrates, sterols, terpenoids, phenylpropanoids, flavonoids, and polyacetylenes [47, 48]. A new compound, 7-phenyl-hepta-4,6-diyne-2-ol, and 20 known compounds-1-phenyl-hept-5t-ene-l,3-diyne, 1-phenyl-hepta-1,3,5-triyne, 2-phenyl-ethanol, linolenic acid, methyl linolenate, ethyl linolenate, 2-butoxyethyl linolenate, α-tocopherylchinon, linoleic acid, 2-butoxyethyl linoleate, 2-butoxyethyl oleate, 2-butoxy ethanol, ethyl linoleate, phytol, phytenic acid, squalene, ß-sitosterol, stigmasterol, 5 α-stigmasta-7-en-3ß-ol and 5 α-stigmasta- 7,22-dien-3ß-ol have been isolated in a study [49]. Several flavonoids, like chalcones okanin and butein along with quercetin 3-O-glucoside, the flavones luteolin and apigenin, have been reported earlier [50].

#### Antiviral activity

Hot water extract of *Bidens pilosa* revealed significant virucidal activity by inhibiting the replication of HSV-1 and HSV-2 [51]. In another experiment, aqueous extract of the plant showed potent antiviral activity against HSV-1 and HSV-2 by inhibiting plaque formation and by blocking binding of virus to host cells and penetration of virus into cells [52].

In different studies, it has been reported that centaurein **(6)**, linoleic acid **(7)**, 3,5-Di-O-caffeoylquinic acid **(8)**, 4,5-di-O-caffeoylquinic acid **(9)**, 3,4-di-O-caffeoylquinic acid **(10)**, luteolin **(11)** isolated from plants have significant antiviral activities against numerous viruses like influenza, Sendai virus, Sindbis virus, Herpes Simplex, Respiratory Syncytial virus (RSV), flavivirus, and polio viruses [47, 53, 54]. Okanin 4`-glucoside **(12)** and butein **(13)**, present in the plant, obstruct the HIV integrase and HIV-1 protease and the entrance of the severe acute respiratory syndrome coronavirus (SARS virus) into host cells. Caffeic acid **(14)**, chlorogenic acid **(15)**, and 3,4-di-O-caffeoylquinic acid **(10)** acted against HIV and polio virus by inhibiting HIV integrase and polio virus protease suppression [54].

### *Boerhavia diffusa* L. (Nyctaginaceae)

The plant is commonly known as Punarnava and is a perennial herbaceous creeping weed, grows up to 1 m, and native to India. Being a tropical plant, it is widely distributed in India, Nigeria, and other parts of the world [55, 56].

#### Phytochemistry

**Fig. 1:**
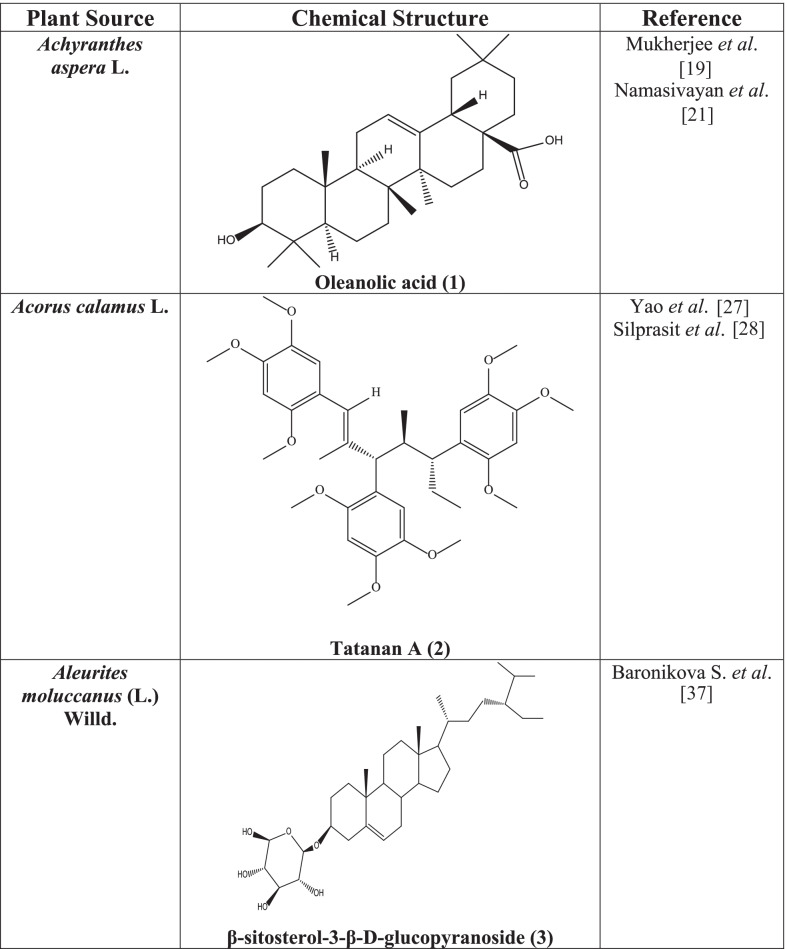

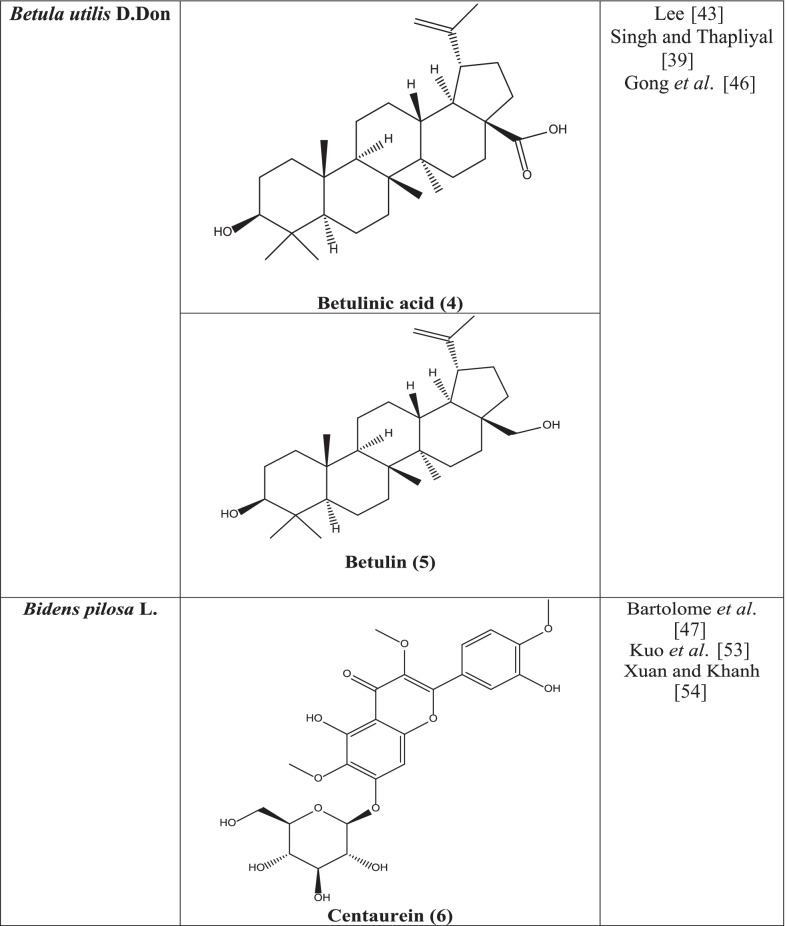

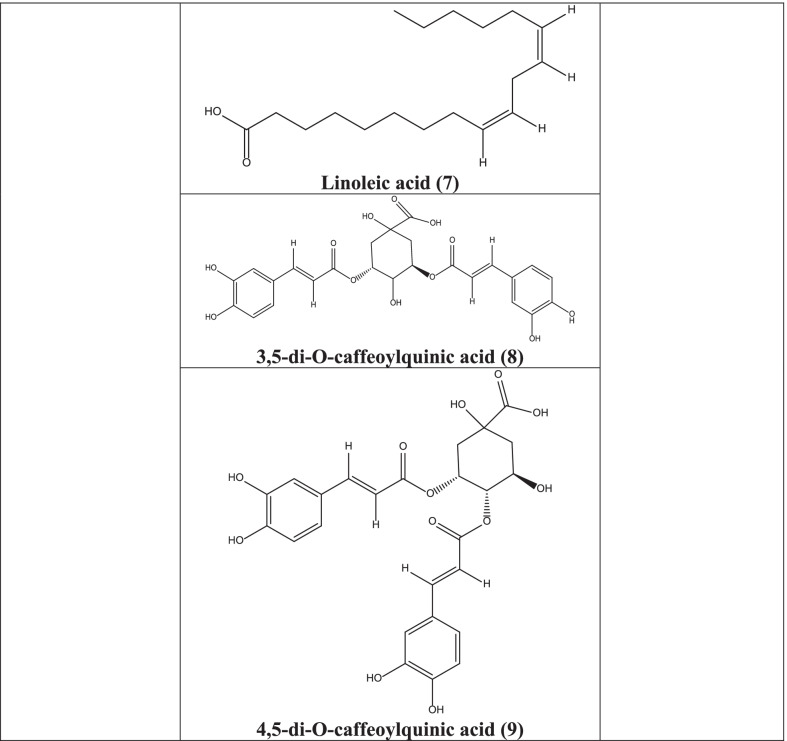

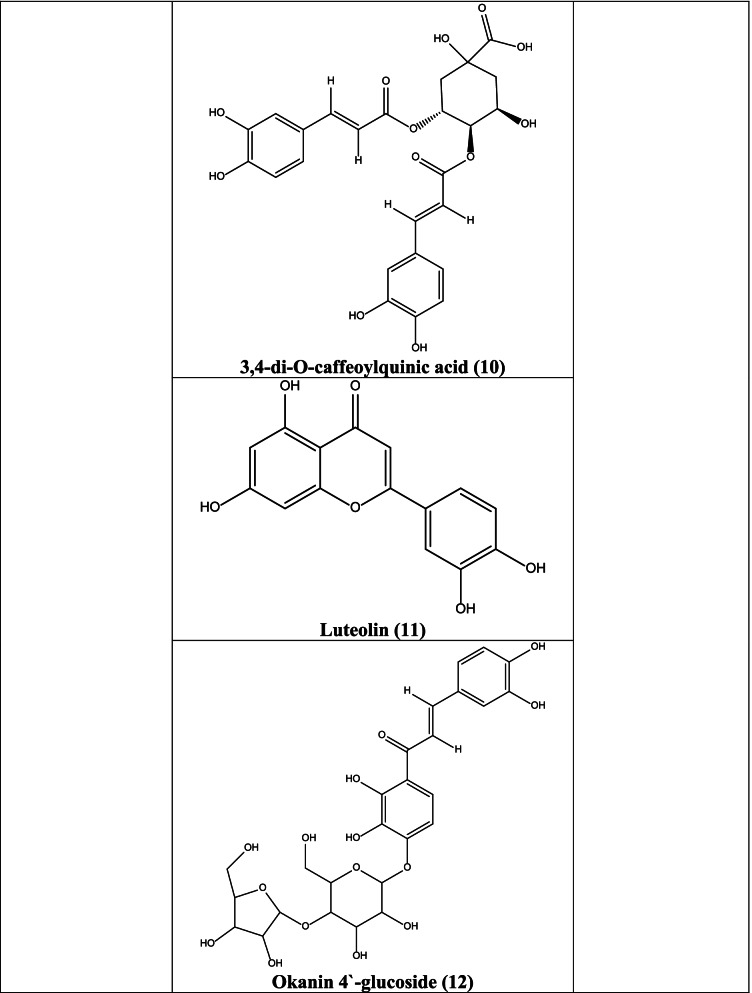

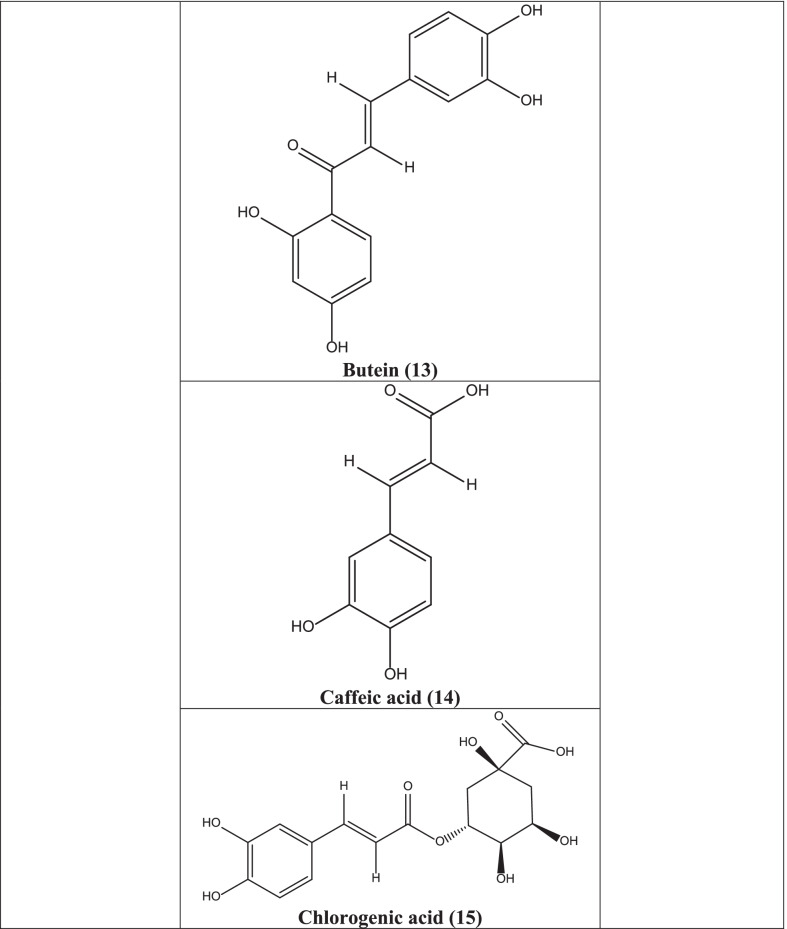

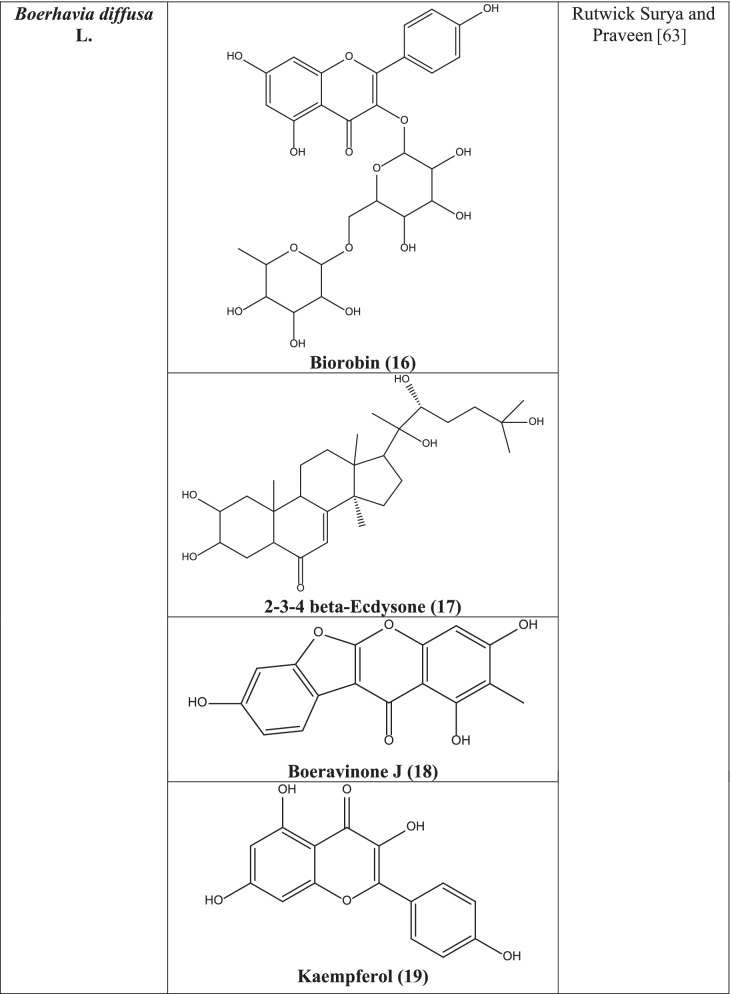

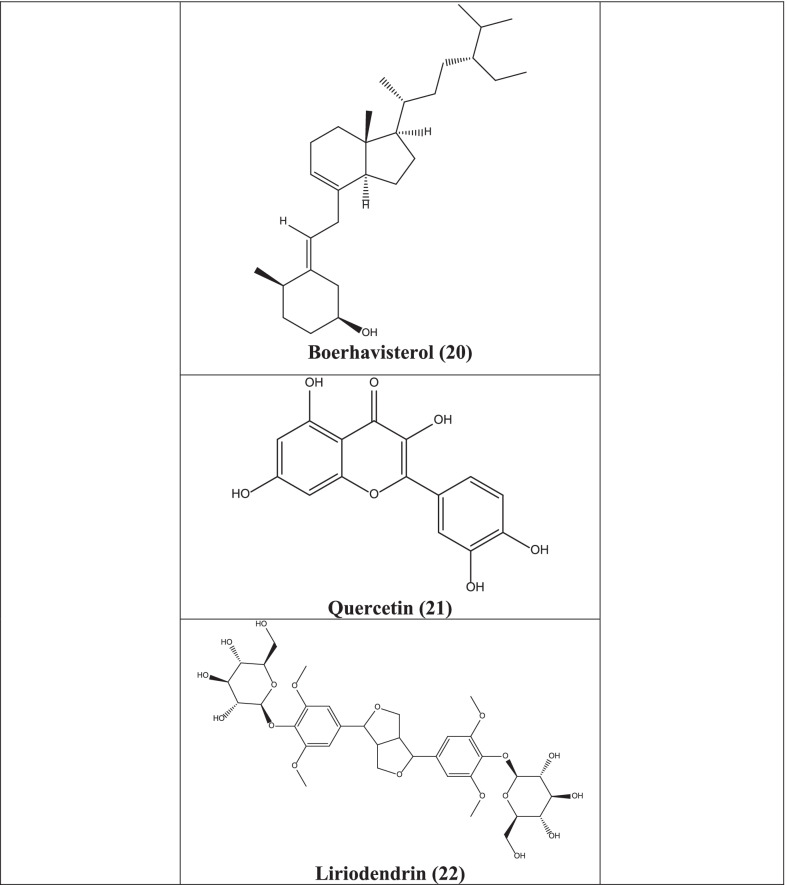

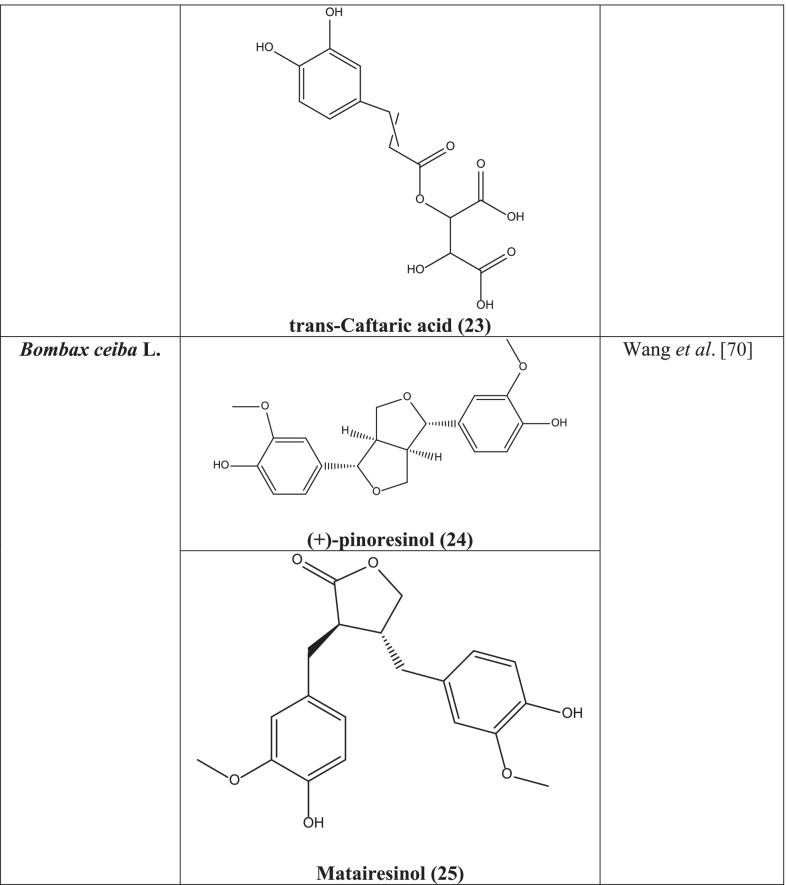

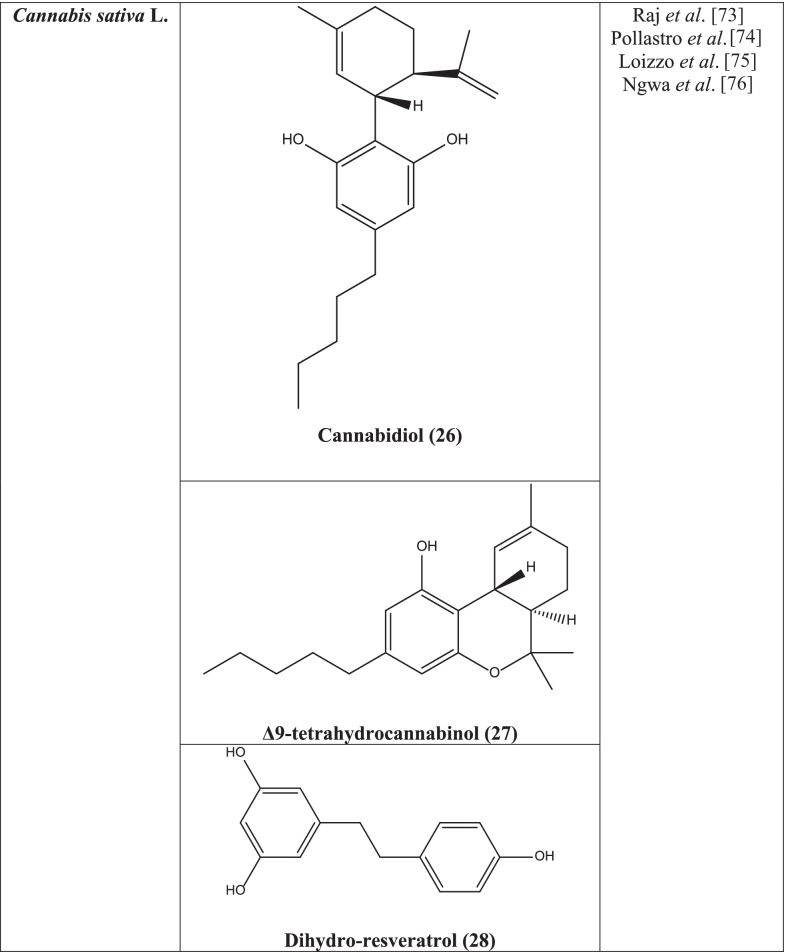

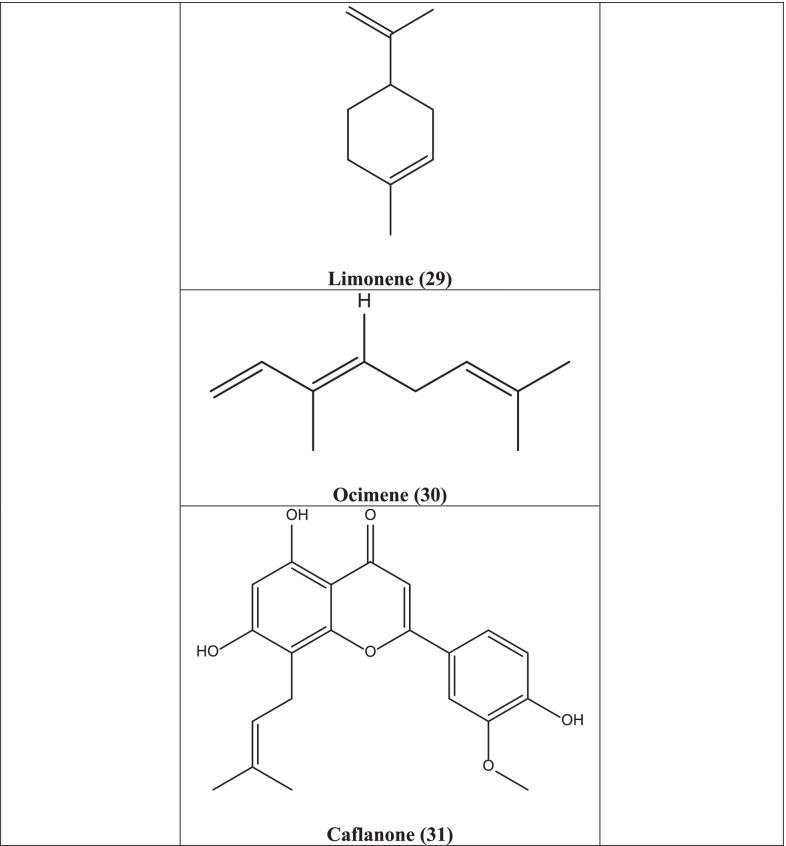

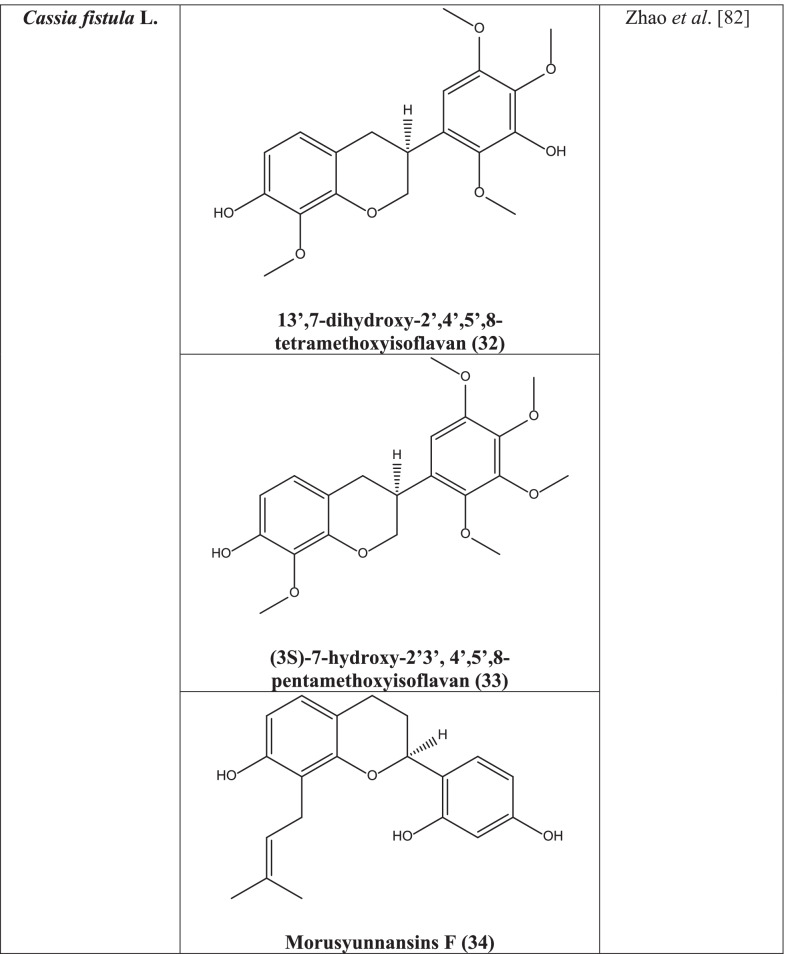

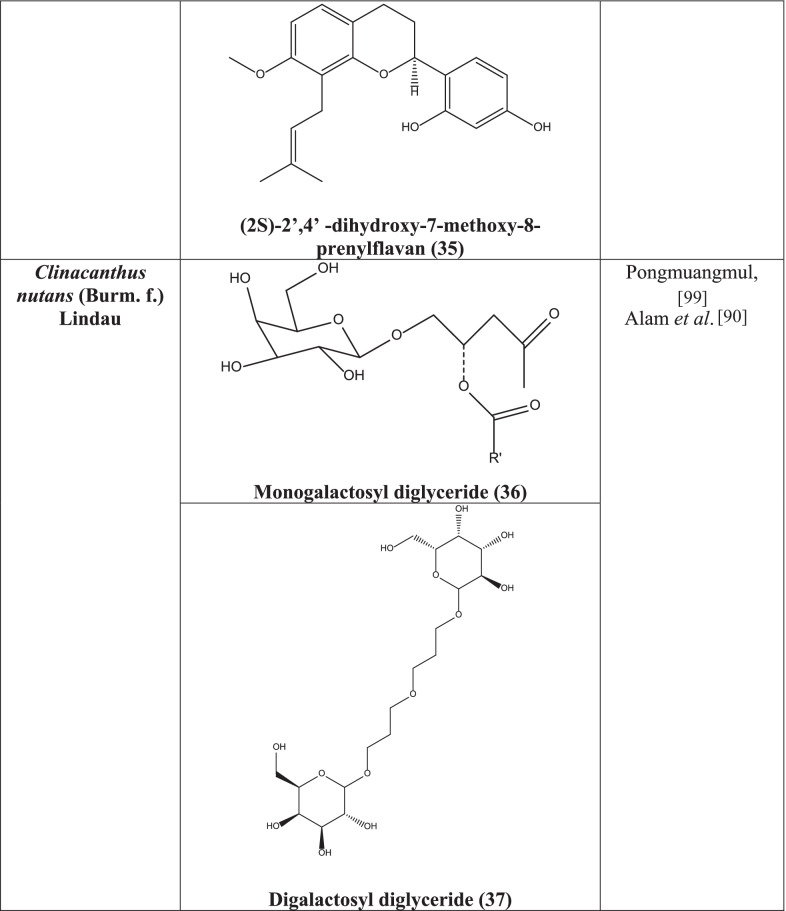

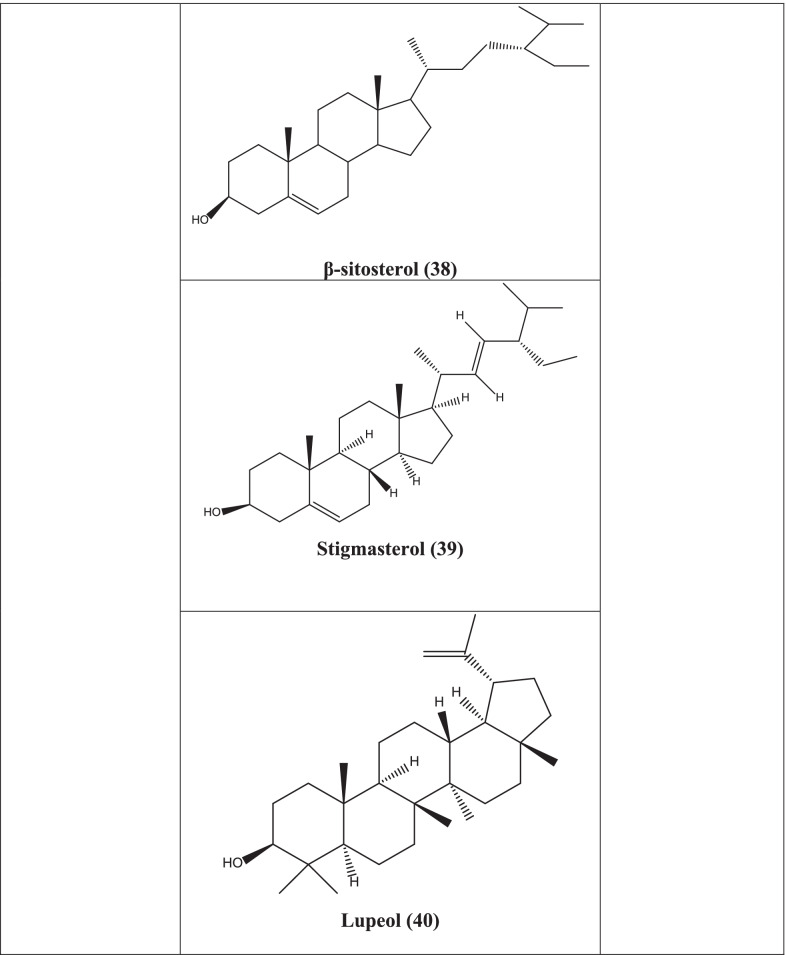

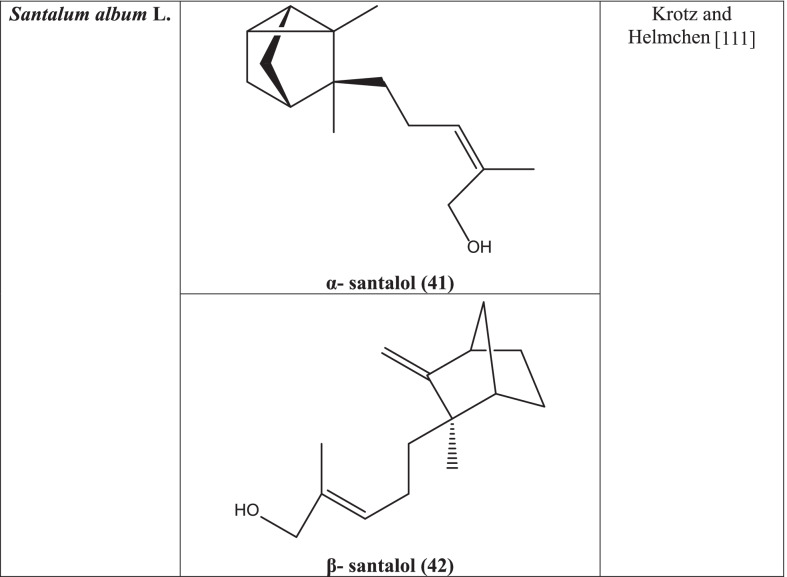
Notable antiviral Asian plants along with their reported phytochemicals

A total of 180 compounds have been isolated from the plant till now. *B. diffusa* is a reservoir of numerous important secondary metabolites like alkaloids, flavonoid glycoside, isoflavonoids (rotenoids), phenolic and lignan glycosides, and steroids (ecdysteroids). Boerhaavone, punarnavoside, kaempferol, 2’-O-Methyl abronisoflavone, quercetin, 3,4-Dihydroxy-5-methoxycinnamoyl rhamnoside, quercetin 3-O-rhamnosyl (1 → 6) galactoside (quercetin 3-O-robinobioside, kaempferol 3-O-robinobioside, eupalitin 3-O-galactosyl (1 → 2) glucoside, eupalitin-3-O-β-D-galactopyranoside, trans-caftaric acid, boeravinones A, B, C, D, E, F, G, H, I, J, 9-O-Methyl-10-hydroxy coccineone, coccineones E, B, 10-demethyl boeravinone C, diffusarotenoid, 6-O-demethyl-boeravinone H, boeravinones M, P, Q, R, S have been isolated from the plant [57]. From the root, 2-glucopyranose-4-hydroxy-5-[P-hydroxyphenyl]-propionyl diphenyl methane was isolated. Many steroids, triterpenoids, proteins, alkaloids, lignins, flavonoids, lipids, carbohydrates, and glycoproteins are mostly found. Punarnavine, ursolic acid, hypoxanthine 9-L-arabinofuranoside, punarnavoside, boeravinone, and liirodendrin have been found. The total plant contains large proportion of proteins and fats. It also comprises 14 amino acids in root, out of which 7 are essential amino acids [58] (Fig. 1).

#### Antiviral activity

*Boerhavia diffusa* have shown excellent effect in inhibiting hypersensitive and systemic hosts against potato virus X [58]. *Boerhavia Diffusa* root extracts were found to have a broad spectrum and strong antiviral activity [59]. The aqueous extract of air-dried roots of *Boerhavia Diffusa* shows broad-spectrum antiviral activity against four viruses- Tobacco Mosaic Virus (TMV), Sunnhemp Rosette Virus (SRV), Gomphrena Mosaic Virus (GMV), and Tobacco RingSpot Virus (TRSV) [60]. In another experiment, glycoprotein from *Boerhavia diffusa* roots acts precisely on virus (es), when it was combined with virus inoculum and incubated in vitro [61, 62].

Biorobin **(16)**, 2–3-4 beta-Ecdysone **(17)**, boeravinone J **(18)**, kaempferol **(19)**, boerhavisterol **(20)**, quercetin **(21)**, liriodendrin **(22)**, and trans-caftaric acid **(23)** present in the plant showed effective docking and subsequent inhibition of SARS-CoV-2 protease in a computer aided docking stud**y** [63].

### *Bombax ceiba* L. (Bombacaceae)

The plant is commonly known as Simul and is a huge deciduous tree with big trunk and spreading crown. Being native to India, it is widely distributed in Pakistan, Vietnam, and China [64, 65].

#### Phytochemistry

All portions of the plant possess β-sitosterol and its glucosides. Lupeol, hentriacontanol, hydroxycadalene, hentriacontane have been found in flowers, bark, seed, and rootbark. The seed oil produces myristic, arachidic, linoleic, oleic, and palmitic acids. Seeds possess carotenes, ethylgallate, n-hexacosanol, and tocopherols; the gum has gallic and tannic acids, yielding D-galactose, D-galacturonic acid, L-arbinose, and D-galactopyranose. Fresher roots contain more sugars (galactose and arabinose) and peptic bodies than the mature ones. Alkaloids, flavonoids, glycosides, proteins, amino acids, and coumarins have been isolated from alcoholic and water extracts of flowers of *B.ceiba* [66] and have been reported to contain the β-D glucoside of β-sitosterol, hentriacontane, free β-sitosterol, hentriacontanol, kaempferol, bits of an essential oil, and quercetin. Shamimin, a newly found flavanol, has been isolated from the ethanolic extract of the plant. 2-hexyl-7, 8-dimethyl-1, 4-naphthaquinone, named ceibanaphthaquinone and lup-20 (29) en-3b-ol, named BC-1 have also been identified from the stem bark [67] Eleven compounds were isolated and identified as squalene, taraxerone, taraxeryl acetate, beta-sitosterol palmitate, 4-methyl stigmast-7-en-3-ol, taraxerol, 6-O-palmitoylsitosteryl-D-glucoside, 1H-indole-3-carboxylic acid, 12beta-hydroxyl-pregnane-4, 16-diene-3, 20-dione, loliolide and 5-(hydroxymethyl) furfural from the plant [68].

#### Antiviral activity

Aqueous-methanolic extract of the plant showed anti-HSV activity in a study [69]. A new lignan bombasinol A along with 4-(4-(3,5-dimethoxyphenyl) hexahydrofuro [3,4- c] furan-1-yl)-2-methoxy-phenol, (+)-pinoresinol **(24)**, 5,6-dihydroxymatairesinol, and matairesinol **(25)** showed anti-Hepatitis B virus by giving showed repressive activity against HepG2 cell lines [70].

### *Cannabis sativa* L. (Cannabaceae)

It is also known as hemp and is an annual herb. Being indigenous to Central Asia, the plant is well grown in Asia, Europe, and China [71]

#### Phytochemistry

More than 538 compounds of different classes have been isolated from the plant till now, and the classes include terpenoids, hydrocarbons, cannabinoids, sugars, and related compounds, non-cannabinoid phenols, nitrogenous compounds, fatty acids, flavonoids, simple acids, simple ketones, amino acids, simple esters and lactones, simple aldehydes, proteins, glycoproteins, steroids, enzymes, pigments, simple alcohols, vitamin (vitamin K) [71]. Cannabisin A, Cannabisin B, Cannabisin M, p-hydroxybenzaldehyde, 3,3′-demethyl-grossamide, Cannabisin F, Cannabisin G, adenosine, N-trans-caffeoyloctopamine, N-trans-caffeoyltyramine, N-trans-coumaroyltyramine, Cannabidiol **(26)**, N-trans-feryroyltyramine, N-trans-coumaroyloctopamine, 4-[(E)-p-coumaroylamino]butan-1-ol, (S)-N-(2-(4-hydroxyphenyl)-2-methoxyethyl)cinnamamide, trans-ferulic acid-4-O-β-D-glucopyranoside, sucrose, and 4-hydroxy-3-acid have been isolated from the plant in an experiment [72].

#### Antiviral activity

Cannabidiol **(26)** and Δ9-tetrahydrocannabinol **(27)** isolated from the plant showed more potency against SARS-CoV-2 virus compared to the reference drugs lopinavir, chloroquine, and remdesivir [73]. Dihydro-resveratrol **(28)**, a metabolite of trans-resveratrol, isolated from *Cannabis* has antiviral activity [74]. Terpenes like limonene **(29)** and ocimene **(30)** which have been found from the plant have also been reported to reveal antiviral activity [75]. A 2020 study confirmed that a small antiviral flavonoid molecule, caflanone **(31)** has selective action against the human coronavirus hCov-OC43 (COVID-19) disease [76].

### *Cassia fistula* L. (Caesalpiniaceae)

*Cassia fistula* is a medium-sized deciduous plant, grows up to 24 m in height, and being native to India, the plant is well distributed all over Bangladesh, Pakistan, and West-China [77].

#### Phytochemistry

Numerous secondary metabolites have been isolated from the plant till now, among which the most important classes are glycosides, proanthocyanidins, flavonoids, essential oils, polyphenols, and terpenoids [78]. Sennosides A & B, anthraquinone glycosides, rhein and its glucoside, aloin, formic acid, barbaloin, butyric acid and their ethyl esters and oxalic acid, presence of pectin and tannin are also reported to be isolated from pulp of the pod. Seeds contain free amino acids and galactomannan free sugars, flowers contain kaempferol, ceryl alcohol, rhein, and a bi-anthraquinone glycoside, fistulin. Leaves produce free rhein, its glycosides- sennosides A & B. β sitosterol and its β-D glucoside and aurantiamide acetate have been isolated from flowers. The roots possess betulinic acid, 7-methylphyscion, and β sitosterol. The stem bark contains two flavonol glycosides, 5,7,4’-trihydroxy-6,8,3’-trimethoxyflavone-3-O- α-L-rhamnosyl (1 → 2)-O-β-D-glucopyranoside and 5,7,3’,4’-tetrahydroxy-6, 8-dimethoxyflavone-3-O-α-arabinopyranoside [79].

#### Antiviral activity

Aqueous hot extract of pods of *Cassia fistula* showed dose-dependent antiviral activity against Infectious Bovine Rhinotracheitis (IBR) virus which is a component of Herpes virus group [80]. In another study, plant extract substantially stimulated IFIT_1_ antiviral protein expression indicating anthraquinones as prospective agonistic compounds for inducing the innate immune system to cure viral infections [81].

Two new flavonoids, fistula flavonoids B and C isolated from the bark and stem of the plant showed high and 3’,7-dihydroxy-2’,4’,5’,8-tetramethoxyisoflavan **(32)**, (3S)-7-hydroxy-2′3’, 4’,5’,8-pentamethoxyisoflavan **(33),** morusyunnansins F **(34)**, (2S)-2’,4’ -dihydroxy-7-methoxy-8-prenylflavan **(35)** showed moderate anti-TMV activity in a study [82].

### *Celastrus paniculatus* Willd. (Celastraceae)

This is an unarmed woody climbing shrub. Being native to India, the plant is widely distributed in Taiwan, Australia, China, Indonesia, Laos, Malaysia, Cambodia, Myanmar, Sri-Lanka, Thailand, Nepal, Vietnam, and many of the Pacific islands [83].

#### Phytochemistry

Malkanguniol, celapanine, malkangunin, celapanigin, sesquiterpene polyol ester, celastrine, celapagin, dihydroagarofuran sesquiterpenoids, paniculatine, celastrol, zeylasterone, zeylasteral, acetic acid, pristimerin, tetracasanol, benzoic acid, oleic, palmitic, linoleic, linolenic, stearic, crude lignoceric acid, and sterol have been isolated from the plant so far [83]. Paraffinic hydrocarbons, β-amyrin, β-sitosterol, and a pentacyclic triterpene diol paniculatadiol were isolated from the non-saponifiable fraction of the CP seed oil. The triterpene diol was assigned structure as olean-12-ene-3β, 29 diol. A new sesquiterpene polyol ester characterized as 1α, 6β, 8βtriacetoxy-9β-benzoyloxydihydro-beta-agarofuran, with the three known compounds: angulatueoid C, 1α, 6β, 8α-triacetoxy-9αbenzoyloxydihydro-beta-agarofuran, and 1α, 6β, 8β, 14-tetraacetoxy-9α-benzoyloxydihydro-betaagarofuran, was isolated from the carbon tetrachloride (CCl_4_)-soluble fraction of *Celastrus paniculatus* methanolic extract of seed [84].

#### Antiviral activity

The plant extract has been reported to use against Bronchitis caused by influenza virus, adenovirus, coronavirus, rhinovirus, and Respiratory Syncytial Virus (RSV) [85].

### *Cinnamomum cassia* L. (Lauraceae)

*Cinnamomum cassia* is widely cultivated in China. Dry bark from this plant has been utilized as folk medicine and spice used in daily diet. It is used in the treatment of inflammation, tumor, pyretic, stomachic. It works against influenza virus or microorganism, and it is also used as an analgesic [86].

#### Phytochemistry

Several compounds were isolated from the plant such as cinnamic acids, cis-4-hydroxy-4-methhoxylexamedhide, coumarin, 4-dihydroxy-5-cyclohexenone. These compounds have been originally isolated from these genus plants [87].

#### Antiviral activity

Cinnamon bark extract and silver nanoparticles prepared from the plant showed promising activity against avian influenza virus subtype H_7_N_3_ in Vero cells while showing no significant toxic effect on the cell [88]. Hot water extract inhibits Human RSV from attaching to airway epithalia. It also prevents internalization and syctium formation of the virus [89].

### *Clinacanthus nutans* (Burm. f.) Lindau (Acanthaceae)

*C. nutans* is a perennial herb that can grow up to 1 m tall with young branches and cylindrical, striate, and glabrescent stems. The plant is widely distributed in Indonesia, Malaysia, Vietnam, Thailand, and China [90, 91]. It is also known as phaya yoin in Thailand, dandang gendis in Java, Sabah snake grass [92].

#### Phytochemistry

A wide spectrum of phytochemicals has been isolated from *Clinacanthus nutans* including flavonoids, glycosides, glycoglycerolipids, cerebrosides, and monoacylmonogalactosyl glycerol, monogalactosyl diglyceride **(36)**. Shaftoside, β-sitosterol **(38),** stigmasterol **(39)** and lupeol **(40)** are a few of notable phytocompounds which have been isolated from hexane fraction of the leaves of *C. nutans* [93]. Also, cycloclinacoside A, cycloclinacoside A_2_, clinacoside A, clinacoside B, clinacoside C, triacetylcycloclinacoside A_2_ were isolated from stem and leaves extract along with some well-known C-glycosyl flavones such as isomollupentin 7-*O*-*b*-glucopyranoside, vitexin, isovitexin, orientin, schaftoside, and isoorientin [94]. Similarly, 13-hydroxy-(13-S)-phaeophytin b, pupurin-18-phytyl ester, and phaeophorbide-a have been isolated from hexane and chloroform soluble fraction [95]. In another experiment, clinamides A, clinamides B, clinamides C, and 2-cis-entadamide A, sulfur containing compounds along with entadamide A, entadamide C, and trans-3methylsulfinyl-2-propenol have been isolated from aerial parts ethanolic extract of the species [96].

#### Antiviral activity

80% ethanol extract of *C. nutans* also found effective against dengue virus [96]. According to [97], ethanolic extract of *C. nutans* can also show inhibitory action against Yellow Head Rhabdovirus (YRV) in black tiger shrimp model [97]. Ethyl acetate extract of the leaves has shown significant antiviral activities against HSV type 1 strain F [98].

*C. nutans* contains monogalactosyl diglyceride **(36)** and digalactosyl diglyceride **(37)** which have shown antiviral potentials against HSV-1 and HSV-2 by plaque reduction assay [99]. In another study, hexane, dichloromethane, and methanol extracts of leaves showed significant antiviral activity against HSV-1 and HSV-2. β-sitosterol **(38),** Stigmasterol **(39)**, lupeol **(40)** isolated from the plant showed same effect probably by interfering with the virion envelope configurations or masking viral glycoproteins, which are crucial for adsorption and entry into host cell [90].

A topical formulation of *C. nutans* extract was prepared, and its effect on 51 patients with Varicella-Zoster virus (VZV) infection examined through a randomized, placebo-controlled trial. The result was promising without showing any side effects [90]. A cream made of extracts of the plant faced some successful clinical trials in the treatment of Herpes Genitalis and Herpes Zoster infection [98].

### Eugenia malaccensis L. (Myrtaceae)

(Synonym: Syzygium malaccense) *Eugenia malaccensis,* also known as Malay Apple or Jamaican guava, is a species of flowering tree that is indigenous to Malaysia, Indonesia, and southern Vietnam [100]

#### Phytochemistry

5,7,3′,4′,5′-Penta-hydroxy-flavonol, 5,7,3′,5′-tetra-hydroxy-4′-methoxy flavonol, 3,4,5-tri-hydroxybenzoic acid, and 3-acetyl-urs-12-en-28-oic acid have been isolated from the leaves and stem bark of *Eugenia malaccensis* [101]. Seeds of *E. malaccensis* also contain a novel lactin (EmaL) (Brustein et al. 2012).

#### Antiviral activity

Aqueous extract of *Eugenia malaccensis* shown antiviral potentials against HSV-1 and 2, as well as Vesicular Stomatitis virus. It was also discovered to suppress the classical complement system, implying that it has an immunological foundation for its in vivo effect. Furthermore, extracts obtained from *Eugenia malaccensis* bark were found to inhibit viral growth at low virus titers [34].

### *Pipturus albidus* A.Gray ex H.Mann (Urticaceae)

The plant, commonly known as Mamaki, is a large shrub or infrequently small tree with slightly hairy stems and 30 feet high. Its territory involves primarily humid and well drained soils. The plant being native in the Hawaiian Islands is being cultivated in Asia and Africa. Herbal tea can be made from the leaves of the plant [102].

#### Phytochemistry

In past studies, catechins, chlorogenic acid, and rutin were found in the leaves of *Pipturus albidus* [103]. It has also been reported that the leaves of this plant contain fat, protein, ash and fibers [102].

#### Antiviral activity

Aqueous extract of *Pipturus albidus* exhibited antiviral effects against the HSV-1 and 2 as well as the vesicular stomatitis virus. It can reduce viral growth at low viral titer [34]. According to a study conducted by [104], *P. albidus* has a very broad spectrum of antiviral potentials [104]. Besides, the development of HIV was inhibited by aqueous extract of *P. albidus* leaves [34].

### *Pluchea indica* (L.) Less. (Asteraceae)

*Pluchea indica* is a perennial shrub plant having small branches (0.5–2 m tall). It is widely distributed in the seaside line of Thailand, Malaysia, Taiwan, Indonesia, India, Mexico, and Australia. It is popularly known as Beluntas (Bahasa), Kuo bao ju (Chinese), Munjhu rukha or Kukrakonda (Bengali) and Indian marsh fleabane or camphorweed [105].

#### Phytochemistry

Several phytochemicals were found from ethanol–water extract of aerial part of *Pluchea indica* including 3,4-dihydroxy benzaldehyde, (3″R)-pluthiophenol, (3″R)-pluthiophenol-4″-acetate, 3″-ethoxy-(3″S)-pluthiophenol, 3″-ethoxy-(3″S)-pluthiophenol 4″-acetate, vanillin, 3,4-dihydroxy-5-methoxybenzaldehyde, syringicaldehyde, dibutylphthalate, ethyl caffeate, 2,3-dihydroxy-1-(4-hydroxy-3-methoxyphenyl)- propan-1-one, trans-coniferyl aldehyde, esculetin, threo-2,3-bis(4-hydroxy3-methoxyphenyl)-3-ethoxypropan-1-ol, erythro-2,3-bis(4-hydroxy-3-methoxyphenyl)-3- ethoxypropan-1-ol, (+)-isolariciresinol, 9-diepoxylignane, (+)-9′-isovaleryllariciresinol, caryolane1,9β-diol, (8R,9R)-isocaryolane-8,9-diol, clovane-2α,9β-diol, valenc-1(10)-ene8,11-diol, fraxinellone, stigmasterol, methyl 9-hydroxynonanoate, triethyl citrate, 9,12,13-trihydroxyoctadeca-10(E),15(Z)-dienoic acid, pinellic acid, adenosine, etc. [106]. In another study, few more bioactive phytochemicals were reported such as dimethyl sulfoxide, 1-propanol, 2-methyl, butanal, 3-methyl, butanal,2-methyl, furan,2-ethyl, 1-butanol,2-methyl, hexanal, 3-hexen-1-ol (z), 1-hexenol, santolina triene, bicyclo[3.1.0]hex-2-ene, 2-methyl, 1 s-alpha-pinene, 3-cyclohexen-1-ol,4 methyl-1-(1-methylethyl), benzaldehyde, 1-octen-3-ol, bicyclo[3.1.0]hexane, 4-methylene, 5-hepten-2-one,6-methyl, bicyclo[3.1.1]heptane,6,6-dimethyl, bicyclo[4.1.0]hept-2-ene,3,7,7,trimethyl, furan,2-penthyl, 3-hexen-1-ol,acetate, 1,3-cyclohexadiene,1-methyl-4-(1-methylethyl), benzene,1-methyl-2-(1-methylethyl)-limonene, 1,4-cyclohexadiene,1-methyl-4-(1-methylethyl), bicyclo[4.1.0]hept-2-ene,3,7,7,trimethylnonanal, p-menth-1-en-8-ol, benzoic acid,2-hydroxy-methyl ester, 2,6-octadien-1-ol,3,7 dimethyl-(z), 3,6-octadien-1-ol,3,7-dimethyl-(z), 2,6-octadenal,3,7 dimethyl,(z), 2,6-octadien-1-ol,3,7-dimethyl-(e), 2,6-octadienal,3,7-dimethyl-(e), naphthalene, etc., were obtained from leaves of *Pluchea indica* [107].

#### Antiviral activity

Aqueous extract of the leaves of *Pluchea indica* was revealed to have therapeutic properties for antiviral efficacy against HIV-1 [34, 108].

### *Santalum album* L. (Santalaceae)

This plant, an evergreen tree, is commonly known as white sandalwood or Indian sandalwood, and is an evergreen tree usually growing up to 20 m reaching with a thickness of 2.4 m with slender wilting twigs. The plant is well distributed in India, China, Sri Lanka, Indonesia, Malaysia, the Philippines, and Northern Australia [109].

#### Phytochemistry

Three new neolignanes and benzoic acid derivatives were found from the *Santalum album* after purification by the chromatographic technique of the ethyl acetate-soluble portion of the methanolic extract. Essential oil from the *Santalum album* was derived from steam distillation which is known as sandalwood oil [110]. The plant is a precious source of volatile oils. This essential oil contains 90% sesquiterpene alcohol of which around 60% is composed of α-santalol **(41)** and 25% is β-santalol **(42)** [111]. Epi-cis-beta-santalol, alpha-trans-bergamotol, cis-beta-santalol, cis-alpha-santalol, cis-nuciferol, α-bisabalol, β-curcumen and other sesquiterpens alcohols as epi β-santalol, γ-curcumen-12-ol, cis-lanceol and trans-farnesol were recorded [112, 113].

#### Antiviral activity

Sandalwood oil of the *Santalum album* has anti-viral activity. Sandalwood oil can inhibit the replication of Herpes Simplex-1 (HSV-1) & Herpes Simplex-2 viruses (HSV-2), the antiviral property was found dose-dependent, and it was more effective against HSV-1 [114].

β-santalol of sandalwood oil has shown inhibitory activity against influenza A/HK (H_3_N_2_) virus by interfering the mRNA synthesis [115]. In another study, α and β-santalol of sandalwood oil were observed to be effective against HPV (Human papillomavirus) [116].

### *Scaevola taccada* (Gaertn.) Roxb. (Goodeniaceae)

(Synonym: *Scaevola sericea*) It is an evergreen shrub commonly known as beach naupaka, fan flower, beach cabbage, umbrella tree, Merambong (Malay), Naupaka Kahakai (Hawaiian), Ngahu (Tongan), Ruk ta-lay (Thai), and Magoo (Divehi). The plant is salt tolerant and well distributed in beach scrubland around the tropical Indian Ocean, the Arabian Sea, and tropical Islands of the Pacific [117].

#### Phytochemistry

According to previous studies, the plant contains chlorogenic acid, scaevolin, saponins, terpenoids, dimethyl acetal, cantleyoside, glycosides, lipids, alkaloids, loganin, steroids sylvestroside-III, etc. [118]. Gas liquid chromatographic analysis of the leaves of the plant *S. tacadda* revealed that it contains series of alkanes ranging from C_14_-C_29_, stigmastrol, cholesterol, campesterol, α- amyrin as triterpene, β-sitoserol [119]. A new compound, scataccanol in addition with 10 known compounds, including five coumarins, marmesin, ent-ammirin, xanthyletin, nodachenetin, and umbelliferone, two iridoids; loganetin and 6-hydroxy-7-methyl-1-oxo-4-carbomethoxyoctahydrocyclopenta[c]pyran, a benzaldehyde derivative; 4-formylsyringol, a cinnamoyl ester; 2-(4-hydroxyphenyl 3-(3,4-dihydroxyphenyl-2-propenoate and a lignan; matairesinol have been isolated from the plant in a study [120].

#### Antiviral activity

Study found that leaves extract of *Scaevola tacadda* was found to be active against vesicular stomatitis, HSV-1 and HSV-2 [117, 118]. It was also found that *S. tacadda* has activity against Human Immunodeficiency virus (HIV) [119].

## Conclusions

Asia has the richest flora of the earth’s seven continents. The region is a substantial source of countless pharmacologically important phytochemicals among which many contain potential antiviral compounds. It is very much possible that the isolation of active constituents from these plants will lead us to the development of more effective antiviral treatment approaches, especially in this era of the emergence of new virus variants. Based on the knowledge from this review article, the regions of Asia should be explored further for discovering valuable phytoconstituents from the plants to develop effective drugs against dreadful diseases caused by viruses.

## Data Availability

Not applicable.

## Abbreviations

AIDS: Acquired Immunodeficiency Syndrome
COVID: CoronaVirus Disease
HSV: Herpes Simplex Virus
HPV: Human papilloma Virus
NDV: Newcastle Disease Virus
AIV: Avian Influenza Virus
HIV: Human Immunodeficiency Virus
YRV: Yellow Head Rhabdovirus
VZV: Varicella-Zoster virus
TMV: Tobacco Mosaic virus
SRV: Sunnhemp Rosette Virus
GMV: Gomphrena Mosaic Virus
TRSV: Tobacco RingSpot Virus
SARS: Severe Acute Respiratory Syndrome
IBR: Infectious Bovine Rhinotracheitis
RSV: Respiratory Syncytial Virus
DENV: Dengue Virus
PPRV: Peste des Petits Ruminant’s Virus
